# Exploring the feasibility of a mental health application (JoyPop^TM^) for Indigenous youth

**DOI:** 10.3389/fpsyt.2023.1269347

**Published:** 2023-10-06

**Authors:** Allison Au-Yeung, Daksha Marfatia, Kamryn Beers, Daogyehneh Amanda General, Kahontiyoha Cynthia Denise McQueen, Dawn Martin-Hill, Christine Wekerle, Tehota'kerá:ton Jeremy Green, The Six Nations Youth Mental Wellness Committee, Tristan Bomberry

**Affiliations:** ^1^Department of Family and Community Medicine, Temerty Faculty of Medicine, University of Toronto, Toronto, ON, Canada; ^2^Department of Biochemistry and Biomedical Sciences, Faculty of Health Sciences, McMaster University, Hamilton, ON, Canada; ^3^Department of Health, Aging and Society, Faculty of Social Sciences, McMaster University, Hamilton, ON, Canada; ^4^Kawenni:io/Gaweni:yo School, Six Nations of the Grand River, Hagersville, ON, Canada; ^5^Department of Anthropology, Indigenous Studies Program, McMaster University, Hamilton, ON, Canada; ^6^Department of Pediatrics, Department of Psychiatry and Behavioural Neurosciences, Faculty of Health Sciences, McMaster University, Hamilton, ON, Canada; ^7^Optentia Research Unit, North-West University, Potchefstroom, South Africa; ^8^Department of Humanities, Faculty of Liberal Arts & Professional Studies, York University, Toronto, ON, Canada

**Keywords:** Six Nations, youth, mental health, resilience, mHealth

## Abstract

**Objective:**

The purpose of the current study was to explore the acceptability and feasibility of a resilience-focused mobile application, JoyPop™, for use with Indigenous youth.

**Methods:**

A Haudenosaunee community-based research advisory committee co-developed the research project, in accordance with OCAP™ principles. Adopting a mixed-method approach, five youths from an immersion school used the JoyPop™ app for four consecutive weeks, as well as completed pre-test questions and weekly usage surveys. Most participants also completed post-test questions and a semi-structured interview. Based on a semi-structured interview protocol, youth responded to questions, and the most common themes were categorized to capture the experience of using the app.

**Results:**

All youth reported a positive impression, used the app daily, found it easy to navigate, and indicated that they would recommend it to a friend. All features were uniformly positively endorsed. There were features that youth used most often (Deep Breathing, “SquareMoves” game, and Art features) and moderately (Rate My Mood, Journaling, and SleepEase). The social connection feature, Circle of Trust, was least utilized, with youth reporting a preference for in-person problem-solving. The drop-down menu of crisis helplines was not used. Youth recommended more gaming options. In terms of cultural resonance, appreciation for the app's use of water sounds in the SleepEase feature was expressed, as was cultural consistency with the “Good Mind” perspective. Recommendations included additional nature sounds, Indigenous design elements, the inclusion of Native language words, and traditional stories.

**Discussion:**

The JoyPop™ app was positively received by Six Nations youth, and ways to ensure its cultural appropriateness were identified. Moving forward, it is recommended that Indigenous designers create a new version with community design co-creation. Additional research with various groups of Indigenous youth is warranted as a pan-Indigenous approach is not recommended.

## 1. Introduction

Indigenous Peoples are a term used to describe the First Peoples of Turtle Island (including Canada and the United States), their ancestors, descendants, and future generations. Within a Canadian context, this term is used to describe three distinct populations: the First Nations, Métis, and Inuit. As of 2021, the population of Indigenous peoples in Canada was at 1.8 million, and it is one of the fastest growing populations, growing by 56.8% from 2006 to 2021 (1). Indigenous peoples are one of the youngest populations in Canada, with 44% of the population under the age of 25 (2). Given disparities in healthcare resources among remote, semi-rural, and rural reserve communities and urban communities, it is important to consider youth sub-populations, especially Indigenous reserve communities where basic living needs (e.g., clean running water) are challenging to obtain. While the greater proportion of First Nations youth live off-reserve in urban and other locales (56%), 44% of First Nations youth live on reserve (3).

The United Nations Convention on the Rights of the Child (UNCRC), accepted by most countries in the world, signals respect for and promotion of a child's right to physical and psychological wellness (4). The United Nations Declaration on the Rights of Indigenous Peoples (UNDRIP) proclaims Indigenous People's rights to maintain and strengthen their distinctive spiritual and cultural relationship with their traditionally owned or otherwise occupied lands, territories, waters, coastal seas, and other resources (5). Health inequities experienced by Indigenous communities stem from centuries of colonization by European settlers, which systematically disrupted traditional community structures, wellness models, cultural practices, and the movement across, utilization of, and stewardship of lands (6, 7). The displacement of Indigenous peoples from their ancestral lands, the disregard of treaties, and the loss of traditional languages have resulted in the persistence of adverse contexts, impacting Indigenous health at a mental, physical, social, spiritual, and cultural level (7–10). For those on reserve, there are intersectional health adversities that impact the optimization of mental health (e.g., resource infractions, ongoing residential school-related trauma, and community violence). In Canada, there has been a persistent over-representation of Indigenous children in the country's child welfare system that serves to limit cultural connectivity (11). In a systematic review, young Indigenous women had an elevated risk of mental health problems (OR = 1.86) and, in the context of maternal depression, small effect sizes were found for increased risk for offspring depression (12, 13). This is significant as parental cultural connectedness was found to be a protective factor for their child's mental health problem risk (12). Despite the fact that First Nations youth face mental health challenges, according to the 2017 Aboriginal Peoples Survey, 48.9% of Indigenous youth reported excellent or very good mental health (3, 12). These authors interpreted these findings in terms of enhanced resilience efforts among Indigenous communities.

Resilience is defined by the United Nations Children's Fund (UNICEF) as “the ability of children, households, communities, and systems to anticipate, prevent, withstand, manage, and overcome cumulative stresses and shocks in ways that advance the rights of every child, with special attention to the most vulnerable and disadvantaged children” (14). To support the development of resilience, it is recommended that programming be developed, and local systems and structures be supported in the planning and delivery of public services (14). In a scoping review of Indigenous youth resilience studies, the broad guiding definition from UNICEF was considered consistent with Indigenous perspectives of interconnectedness and interdependence (15). Considering this perspective, important resilience pathways include connecting to the natural world, learning from animals, inter-generational teaching, and mentoring relationships (15). Others have defined Indigenous resilience as a long healing journey to address multiple, historical, ongoing, and current traumas. Drawing upon decolonization processes, resilience factors for Indigenous youth include opportunities for empowerment, positive cultural identity, and a future orientation (15–17). Resilience programming (e.g., creative art expression and access to Indigenous language immersion education) supports the development of the capacity to respond to and overcome risks (18–20). Given the active elements in traditional ceremonies, including learning from the land, observation, and trial-and-error learning, Indigenous youth seem to gravitate toward educational empowerment through personal expression opportunities to build self-esteem, self-identity, and skillful, healthy relationality (21). For example, in an arts-based qualitative study, three themes emerged: (1) nature as a calming place, particularly in relation to water and bodies of water; (2) nature as a metaphor for resilience, in terms of growth and renewal; and (3) nature as hope, in terms of future opportunities and positive change (22). More research is needed that supports Indigenous youth voicing what resilience strategies are found to be personally helpful, as Indigenous youth may be less likely to seek formal health services because of stigma, discrimination, concerns around anonymity, distrust of governmental services, the availability of culturally relevant and trauma-informed approaches, challenges in identifying signs and symptoms of mental health problems, and difficulty with where or how to seek help (9, 16, 23).

In a national survey of 675 Indigenous youth in the United States, it was found that 78% had regular access to a mobile phone (24). Hence, mobile applications have been identified as an accessible tool to support Indigenous youth resilience, especially in offering accessibility, affordability, and the capacity to address “in-the-moment” needs, without necessarily depending on Internet connectivity (9, 25). Research reveals that youth generally prioritize anonymity and privacy, ease of use (“look and feel”), and interactivity when engaging with mental health applications (26). The unique realities of Indigenous youth, wherein they navigate two worlds of traditional Indigenous culture and modern youth culture, have challenged researchers to identify adaptive frameworks. A two-eyed seeing approach has been popularized to accommodate both Indigenous and Western knowledge traditions (27, 28). Two-eyed seeing is rooted in Indigenous knowledge systems that emphasize non-hierarchical sharing and facilitate a “dialogue” between Western and Indigenous ways of knowing (27, 28). Seeking to harmonize these two distinct viewpoints, this approach recognizes and values the unique strengths and insights that each perspective brings (27, 28).

**Figure 1:**
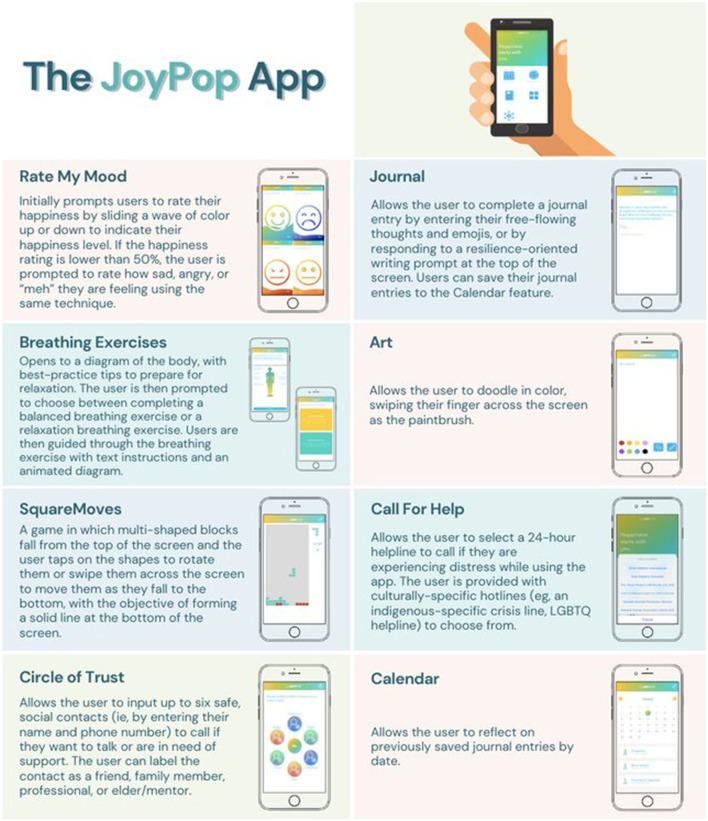
Features of the JoyPop™ app.

The JoyPop™ app (see Figure 1) is an iOS English and French language mobile application designed with the intention of promoting and enhancing resilience among youth (youthresilience.net). While it was initially produced as a potential tool for at-risk youth, particularly those with trauma backgrounds, its focus on positive emotions and actions has the potential to support a broad spectrum of users. App development was supported by professional app developers, researchers, computer sciences and health sciences university students, the Toronto Police Services high school youth leadership group (i.e., Teens Ending Abusive Relationships, T.E.A.R), and practitioners within child welfare, which is described elsewhere (25, 29). The key proposed mechanism of the app is the facilitation of emotion regulation (ER). ER skills (e.g., awareness, self-reflection, labeling, modulation, expression, and management of positive and negative emotions) develop substantially across adolescence with the advent of greater autonomy strivings, more abstract thinking, normative relational challenges (e.g., friendships, romantic partnerships, work relationships, greater autonomy strivings), and changing neurocircuitry (e.g., increased prefrontal “control” and problem-solving) (30, 31). ER reflects the developmental capacity to integrate feelings and thoughts about emotions with actions that are driven by habit, reactivity, or impulsiveness (or reflexivity) or impacted by conscious, effortful responding (or reflectivity). Disrupted ER is central to the experience of anxiety and depression, in terms of managing negative emotions and accessing positive emotions (32). The JoyPop™ app was built on the assumption that resilience is a skill set that can be increased through targeting ER components, specifically skills to dampen physiological reactivity, high negative emotionality and low positive emotionality, and high cognitive load (17, 25, 29). Positivity is enhanced with a focus on positive emotionality (e.g., happiness) and personal agency (e.g., motivational messaging and encouragement for users to attend activities if their mood is rated low) (17, 25). Specifically, ER is addressed: (1) physiologically with diaphragmatic breathing (33) and quality sleep (25); (2) affectively with mood awareness and monitoring (34) and unstructured art expression (35); (3) socially, with close relationship connecting and distress line support (25); and (4) cognitively with journaling with resilience-oriented prompts (36) and focused attention gaming techniques (37).

In evaluating the JoyPop™ app with adolescents who had transitioned to their first year of university, JoyPop™ improved both ER and depression scores, with youth readily adopting the app into a daily or near-daily routine over the study month (29). A qualitative research study also found that the JoyPop™ app was readily adopted into users' daily routines, being seen to positively start and end their day. Users appreciated the opportunities to be expressive with how they were feeling in a variety of modalities. Similar themes were found in a qualitative study by Kim et al. (38), who gathered the perspectives of adults from the Six Nations community to assess the appropriateness of JoyPop™ app for Indigenous youth. These three JoyPop™ studies, however, did not target Indigenous youth to provide an exploration of how the app would be received. The purpose of the current study was to explore the accessibility and feasibility of the JoyPop™ app with Indigenous youth living on reserve. Specifically, we queried the opinions of the app and its features among Haudenosaunee youth of the Six Nations of the Grand River, as well as the youths' reported usage, to explore accessibility and feasibility issues.

## 2. Methods

To provide a methodology overview, this study is a qualitative case study based on phenomenological research. The case study is an appropriate research method for the exploration of a phenomenon within a particular context, including natural setting that is culturally relevant; the case study benefits from the undertaking of the exploration through a variety of data sources (39). Taken together, a potential process and framework may emerge that are useful for further research.

This research was conducted at Canada's largest First Nations reserve, Six Nations of the Grand River (SN), in the Great Lakes region (Ontario, Canada). SN is home to six distinct nations—Cayuga, Mohawk, Seneca, Onondaga, Oneida, and Tuscarora—and is a semi-rural community governed by the Haudenosaunee Confederacy Council and the Six Nations Elected Band Council. With an on-reserve population of 12,849 individuals, and a cumulative band membership of 28,019, this community has engaged in health science research for decades (40). Philosophical paradigms for Indigenous research necessitate the underpinning of Indigenous Nation-specific laws. Embedded within the fabric of this community is Haudenosaunee law, an embodiment of the alliance between nations under the Great Law of Peace, or *Kayannerenkó: wa* (41). One important embodiment of Haudenosaunee values and perspectives is the “Good Mind” and its correlates of acting in a “good way” and in thanksgiving to all beings, such that all come together as one (42). The Good Mind is considered that which keeps a person balanced and in harmony. It reflects an individual point of accountability to oneself, one's clan, one's community, one's nation, and, ultimately, to the natural environment (Mother Earth). The Good Mind is a physical, psychological, and spiritual journey that includes a reflective awareness of thoughts and intentions, and a way of being that is expressed through self-compassion and compassion for other beings (43). These contextual elements highlight the requirement for Western researchers to be invited into the territory (as is the custom among Nations), and the essential learning about culture to appropriately shape the research process, results, and interpretation.

### 2.1. Researcher reflexivity and positionality

All Six Nations authors live and/or work on the reserve and represent the Mohawk, Cayuga, Seneca, and Oneida nations. All have also attended Western universities and/or colleges and are deeply embedded in traditional culture, ceremonies, and language. Indigenous committee members engaged with Western researchers within regular meetings and on an as-needed basis, as well as providing a listing of culture-specific readings. Particularly among Western academics conducting Indigenous-focused research, reflexivity and positionality are key to validating qualitative research findings and promoting trust in co-creation. The first author, AA, is a non-Indigenous student who completed a bachelor's degree in Health Sciences from McMaster University. She now benefits from financial aid resources to attend the University of Toronto. Author DM is a non-Indigenous, South Asian student from McMaster's Department of Biochemistry. Author KB holds both settler and Indigenous ancestry from the Chippewas of Rama First Nation. Her academic background in Health and Aging from McMaster University focuses on culturally relevant youth programming in Indigenous communities. Authors AA, DM, and KB completed a nationally promoted course from the University of Alberta to better understand Indigenous culture and attended group meetings with the Six Nations Youth Mental Wellness Committee. Author CW is a white, non-Indigenous, European ancestry settler, who benefited from Western education in obtaining a Ph.D. (Clinical Psychology). She has worked with Indigenous welfare leaders since the early 2000s and has engaged with Indigenous community members through various publicly funded research projects, including on trauma and resilience among Mi'kmaq youth living on reserve in Nova Scotia, Canada. She worked on water-related research with the Six Nations prior to forming the Six Nations of the Grand River Youth Mental Wellness Committee to specifically guide adolescent research. She attended an online visit through Canada's first residential school in Mohawk territory. Throughout the life of the current project, she was mentored by an Elder from the Six Nations in 1:1 meetings. Reflective practice and positionality discussions occurred in the context of the committee meetings and larger dissemination events.

### 2.2. Participants

The Six Nations of the Grand River Haudenosaunee community identified an appropriate site for youth research, which was a private, on-reserve Mohawk and Cayuga language immersion school that ranged from kindergarten to grade 12. The school's vice-principal and counselor were available to youth, parents, and teachers to respond to inquiries about the research and provide information in the Indigenous language, as needed. The study was introduced to youth with 15-min presentations delivered by a Six Nations research assistant to each grade-eligible class (i.e., grades 7 to 9). Presentations highlighted the features of the JoyPop^TM^ app, the purpose of the study, and the study's timeline. An information sheet and recruitment poster were distributed by the school counselor to all eligible students via their school email. Interested youth provided consent as per guidelines detailed in the ethics portion of this article, and consent was stored separately from data on a password-protected institutional drive.

With the goal of understanding the experience of using the app, a phenomenology approach was undertaken. Generally, this approach requires a sample size between 3 and 25 participants (44). As class sizes were small, the study could have maximally recruited 25 youth. This study recruited a group of five youth participants (2 males; 3 females), from 14 to 16 years old, with one youth at 11 years old. Four youth reported their languages as Mohawk, two as Cayuga, and one youth did not provide a response. All youths participated in the pre-questionnaire and weekly usage questionnaires, and four youths elected to complete the post-test questionnaire and complete an interview.

### 2.3. Data collection

Participants were assigned a study ID code to use in any data collection and asked to complete a pre-test survey. They were then given access to download the JoyPop™ app directly onto school-provided iPads and were instructed to use the JoyPop™ app daily for four consecutive weeks, simultaneously completing weekly surveys. The app itself has onboarding instructions, and there was a 4-min instructional video on app features and use of the app (https://www.youtube.com/watch?v=3LzYTdjZPnU&t=64s) which youth were directed to watch. Following the 4 weeks, youth were asked to complete a post-test survey and offered the opportunity to participate in an individual interview to share their experience with the JoyPop™ app.

### 2.4. Research tools

Prior to using the app, a pre-test survey was distributed, which consisted of questions ranging from open-ended demographic-related inquiries to forced-choice questions (yes, no, or unsure) about lifestyle and closed scales using the 5-point Likert Scale assessing baseline ratings on perceived mental wellness. These were developed in conjunction with the SN Youth Mental Wellness Committee. Weekly surveys were also distributed to track the self-reported frequency of daily app usage; youth were asked how many days within the week they accessed the JoyPop™ app and how frequently per day. A post-test survey was used to query youths' opinions about the app. Questions were similar to the pre-test survey, with additional forced-choice questions about the app's helpfulness and overall impressions. For those who offered to share more feedback, interviews were delivered via Zoom, a video-conferencing tool, using audio only, by a research team member. Each interview was 1 h in length and followed a semi-structured format, with two general topic questions (e.g., Would you please describe what mental wellness means to you?; What does a “good mind” (Haudenosaunee concept) mean to you?). Following these, the interview completed the previously established JoyPop™ interview protocol outlined in Kim et al. (38) wherein the 4-min JoyPop™ introductory video was played and participants were asked for their thoughts on the app's features, first shown in complete and then re-run with pre-specified pauses at the end of each feature description (e.g., After the Breathing Feature description on the video, the video was paused, and youth were asked, “What did you think about the Breathing feature?”). Open-ended questions guided the conversation, and youth were encouraged to bring up new topics as they wished (e.g., “Is there anything else you would like to discuss that we did not ask you about?”), and thoughts about the app (e.g., “Outside of this research study, would you use JoyPop™, why or why not?”). Following the interview, a summary of the discussion points was emailed to each youth for member-checking purposes to ensure the accuracy of the data gathered and description. This process facilitated relinquishing the expert “researcher” role to listen for youth voices (e.g., “I” statements) and reflect on how the voices fit together in terms of similar opinions and where there were unique or divergent opinions, ordering quote segments in terms of similarity based on frequency across youth participants.

### 2.5. Data analysis

Each consultation was transcribed by two research assistants (AA and CM). In the adult study, a coding framework was developed to facilitate content analysis, in which descriptive labels, or codes, are used to capture and summarize themes discussed by the consultants (38). Additional codes were added to ensure youth's questions and perspectives were represented. Content analysis was the selected analysis method as it allows researchers to identify cultural patterns, themes, trends, and other features from verbal, visual, or written data (45). Using the framework, each transcript underwent a separate coding process in a double-blind fashion. Inter-rater reliability scores were calculated to ensure consistency with the coding process, with inter-rater reliability exceeding 85% (46, 47).

### 2.6. Ethics

Several factors were in place prior to conducting this JoyPop™ study with Indigenous youth living on reserve. These included first being invited by the Six Nations of the Grand River Haudenosaunee community to conduct research on JoyPop™, where the app's face validity was seen as a good fit with community emphasis on youth resilience. Our community-based trainee (KCDM) co-presented this research proposal with CW at a Canadian Institutes of Health Research Gender and Wellness Development Fair, which allowed for broader discussion with national Indigenous representatives and youth, on the rationale and structure of the JoyPop™ app and its research. This facilitated the formation of a Western (CW) and Six Nations (KCDM and DMH) research co-leadership to seek funding to develop an approach to app development. The school principal joined the research team as co-applicant and provided support for schools staff to act as project consultants and facilitators. The early partnering with school staff provided important directives in terms of a cautious approach inviting youth to participate in research, using school-based communication channels to first approach parents and guardians, then present at teacher meetings, and finally, conduct in-class presentations to youth. A collaborative plan supported a budget for remuneration (i.e., gift cards directly to youth, and iPads to the school), as well as establishing dedicated school staff as part of a guiding committee.

Having secured funding, a community-based Six Nations Youth Mental Wellness Committee was established to meet regularly. The Committee member remuneration was at $50 per 1 h meeting. Any additional member-specific meetings also occurred at this remuneration level. This committee was composed of the Indigenous Six Nations grantees and broadened to a diverse membership of health services, education, language, and culture expert adults with community leadership roles. Specifically, this committee recommended several procedural approaches: (1) first conducting a qualitative interview study of adult community members who were involved in youth services to gather information about their views about the app, their thoughts about its relevance to youth, and thoughts about cultural adaptations to the app; (2) utilizing the immersion school as a test case site and adhering to the typical school communication procedures (e.g., secure parental section on school's website); (3) maximizing privacy so that data are stored with an ID number and that no app back-end data would be collected; (4) engaging committee members in-depth about the interpretation of findings within a cultural lens; and (5) developing a post-study action plan that included storage of data within the Six Nations site.

School guidelines and committee feedback steered the recruitment process. Interested youth below the age of 16 were encouraged to review a consent form with a parent or guardian, and active guardian consent was required. For those age 16 or above who were interested, the youth was deemed as able to provide their own consent. Youth were remunerated for participation in the questionnaire and interview components via online gift cards ($35 total).

This study followed the OCAP^TM^ (Ownership, Control, Access, and Possession) principle framework, which guides the collection, protection, use, and sharing of First Nations data and ensures caution is taken with youth-specific research (48). Ethics approval was received by Haudenosaunee Confederacy Council, Six Nations Elected Band Council's Research Ethics Committee, and McMaster Research Ethics Boards for consultation interviews and survey data collection (MREB #3728; HIREB #12572). Our advisory committee presented in Mohawk to the longhouse meeting of the Confederacy leadership, and our community-based trainee (DM) co-presented this research idea at a Canadian Institutes of Health Research Gender and Wellness Development Fair, with Indigenous co-leadership, which allowed for focus group discussion more broadly on the rationale and structure of the JoyPop™ app and its research.

## 3. Results

### 3.1. Pre-test, weekly surveys, and post-test

When asked to rate their health on a 5-point Likert scale, youth rated their physical health as good (“good” or “very good”) and had mixed ratings on their mental health (from “fair” to “very good”). In terms of digital technology, all youth reported using social media and communication apps daily over the past 12 months. In terms of health or wellness apps, three of the five youth reported having used them in the past.

Youths were sent an email at the end of the school week to report which days of the week the JoyPop™ app was used. Youth reported that they minimally used the app once a day and maximally three times a day across the 5 days of the school week.

After 4 weeks of using the JoyPop™ app, a post-test was done to determine final impressions, in which four out of five consultants participated. In response to a forced-choice question (yes, no, or unsure), all youth reported that they would recommend the JoyPop™ app to a friend. In response to a question on which feature they used most often, all youth identified using the Tetris-like gaming feature, SquareMoves, most often. Youth reported using all features except the dropdown of crisis helplines and the Circle of Trust feature. In terms of an open-ended question on barriers to using the app, three out of four youths reported that forgetting to use the app interfered with the frequency of their use of the app. It should be noted that the app uses no “push notifications” to prompt use.

### 3.2. Qualitative interviews: main themes

As small number of youth contributed to this study, we do not use any identifiers for quotations from youth. In each section, examples are from different youth. The SleepEase feature, which provides support for getting into a sleep mode, was reported as being used by only one participant and, as such, is not presented below. While the Circle of Trust feature was not used by any youth, it is discussed below because it was the most valued feature *a priori* by advisory committee members.

#### 3.2.1. Mental wellness

Sub-codes and examples are found in Table 1. Common codes, or themes, identified when discussing mental wellness included concepts of positivity/happiness, positive body language, personal hobbies, acts of kindness, and difficulty understanding emotions. All youth described positivity and happiness as important contributors to their mental wellness, and that the app could provide emotional support. Culturally, the youth recognized that a Good Mind had positive emotionality as a key feature and that the app had a positive emotional orientation. Most youths elaborated upon several examples of positive body language (e.g., being active, greeting others, smiling, laughing, movement) that represented mental wellness. Specifically, laughter was noted as a sign of spreading “good energy” around to others. Most youth indicated that personal hobbies were a part of maintaining their mental wellness and making them feel happy. These hobbies ranged from reading to dancing to sports (It is noted that youth are engaged with traditional dance lessons, with an annual Pow Wow held on reserve; furthermore, lacrosse teams and leagues are prominent). One youth noted that “I'm going to say reading, again, because it puts me in an exciting mood, and I'm happy to read.” (It should be noted that the app allowed for the saving of journaling entries within a calendar feature so that youth could revisit and read what they had written).

**Table 1:** Mental wellness: sub-codes and quote examples.

**Parent code**	**Sub-code**	**Example of youth's quotes**
Mental wellness	Positivity/happiness	A good mind is someone who has like just good thoughts positive thoughts all the time and uses kind words all the time just being positive.
	Difficulty understanding emotions	Now that I'm this age I'm more bringing up how I feel and how I deal with my emotions which is why it is really hard for me to describe when I'm happy or when I'm happy and how to express it in a healthy way either so right now I think I'm learning how to express like being happy and being just emotions cause usually I just shove everything down.
	Acts of kindness	“And what are some things that you do to make sure you have a good mind in your day-to-day life?” (interviewer) “Helping people like with their needs or whatever. And like just being kind to your family members and taking care of stuff, and helping whoever.”
	Personal hobbies	Let's see. I'm going to say reading again because it puts me in an exciting mood and I'm happy to read.
	Positive body language	“What are some ways that you express your happy emotions?” (interviewer) “Probably like smile a lot and laugh and if I'm really happy, I jump around, so stuff like that.”
Cultural	Relationships	You could do anything and have a good mind about it you know… Talking to your elders, respect everyone you know… um… I was taught this by an elder at the long house.
	Nature	I like to spend time outside, you know… Be with nature and mother earth just to see all she created for us. Or uh what Creator created for us. All this beautiful stuff we see with your own eyes… he did that all for us you know.

Additionally, all youth indicated that acts of kindness were important to their concept of Good Mind as their own behavior carries responsibility toward community members. Youth expressed that these acts of kindness were rooted in caring for oneself and others. For example, three youth provided the following:

Helping people, like, with their needs or whatever. And like just being kind to your family members, and taking care of stuff, and helping whoever.

Treat people the way you want to be treated, I guess.

A Good Mind is someone who has, like, just good thoughts, positive thoughts all the time, and uses kind words all the time… yeah, just being positive.

Furthermore, all youth expressed difficulty understanding their emotions at times, especially when mixed emotions are involved. Youths reflected that they are still learning to express and understand their own emotions. For example:

Now that I'm this age, I'm more bringing up how I feel and how I deal with my emotions, which is why it is really hard for me to describe when I'm happy or how to express it in a healthy way either. So right now, I think I'm learning how to express, like, being happy and being just emotional, “cause usually I just shove everything down”.

#### 3.2.2. Cultural importance of relationships and nature

When discussing culture, themes identified by all youth included relationships and nature. All the youth mentioned the importance of relationships, and most said that their relationships with family and friends were important for maintaining their happiness and mental wellness.

OK, so what's the visual or thought that brings a smile to your face? Probably my little cousins. And lacrosse. Family dinner.

You could do anything and have a good mind about it, you know… Talking to your Elders, respect everyone you know… um… I was taught this by an Elder at the Longhouse.

All the youth identified that Nature is important to a Good Mind, bringing positivity through relationality (i.e., connected and equal to all living things and having gratitude for creation). Nature-based visuals and sounds, such as the sound of rain, thunder, crickets, and flowing waters, were found to be very positive (It should be noted that there are specific teachings attached to natural occurrences, such as thunder representing grandfather beings). Two youth examples are as follows:

I go into these stages where all I think about is the sound of water, the sound of thunder, the sound of rain and that puts me at peace.

I like to spend time outside, you know… Be with nature and Mother Earth just to see all she created for us. Or, uh, what Creator created for us. All this beautiful stuff we see with your own eyes… he did that all for us, you know.

#### 3.2.3. Mobile application usage

Mobile Application Usage refers to the youths' general usage of any type of mobile application. The most common themes were related to games, social media, and other applications. Most youth stated that they used games to pass time and enjoyed the reward aspects (e.g., collecting coins and customizing avatars). Most youth listed applications for specific information, entertainment, and communication. Some examples are as follows:

For particular reasons, like the weather and stuff…

*Subway Surfer* just passes time. I really like that app because we can just pass time or whatever.

For Instagram, I always go on the explore page, and there are a bunch of quotes slash memes about what people are feeling, and I save them, and really like them because they really have a way of ‘oh, I relate to them.' That's the only way I can express my feelings. So, I really keep those in my main part of why I use Instagram. Also I get book recommendations from there. Snapchat is more just like talking to my friends from like hours away, and talking to friends down here, it's more of like a communicating app for me.

#### 3.2.4. Joypop™

Youth opinions about the features of the JoyPop™ after having used it for 4 weeks were collected. Overall, the ease of using the app and the value of the different activities were consistently positive among all youth. Youth responded to open-ended questions about the app more generally, with comments provided below on aspects of the user experience (e.g., “the look and feel”). Comments about specific features are discussed, first examining features that youth reported using most frequently (Breathing, SquareMoves, Art), followed by those used moderately (Journaling, Rate My Mood), and those used rarely (Circle of Trust). Finally, youths provided their ideas on how to improve the app in terms of aligning with culture.

##### 3.2.4.1. App layout and user experience

Youth were asked about the aesthetics and efficiency of the app layout and designs in terms of the icons used (e.g., color scheme, smiley face on the home screen; the home screen text “Happiness starts with you”; the icon designating features on the activities page, thumbs up visuals with positive affirmation comments, prompts to go to activities from mood ratings, the diagrams supporting body relaxation cues, and sleep hygiene tips that appeared before using the breathing and sleep features, respectively). Overall, youth regarded all these positively, and none noted negative reactions. The icons and symbols were familiar to youth, and the movement across screens was considered easy to navigate, in an intuitive way. Youth did not feel they required additional instruction to use the app (beyond the Circle of Trust, as noted below), and it was an advantage to be able to use it without the need for an Internet connection. Youth liked that the app was simple, without too many features or words, and that the language and words used in the app were easy to understand. Youth comments are below.

What do you think about the colors that are used in the app?' Well for me those are my favorite colors.

I think the [icons] are pretty cool, and you can like understanding what the app part is of it. So, like, for example, the art has a little art symbol on it so you know that's the art, and like the breathing has the face breathing and stuff so like, yeah.

Um, well, it's just a really fun app to use and, like, it makes all my boredom go away. Like, if I'm at my brother's lacrosse game on the rez, I can just pop that on and use it.

##### 3.2.4.2. Breathing feature

All youth found the breathing animation to be a novel activity and appreciated that it uses steps to guide the breathing cycles and that there was more than one breathing activity. No youth reported any negative reactions; the most common sub-code found in the interviews was *Breathing Feature: Positive Reaction*. All youth reflected that, while they had been aware of breathing and how emotionality affects breathing, they had not done specific breathing exercises before. Youth noticed an immediate positive impact from focusing on their breathing. Youth found the breathing exercise options useful for relaxation and for regaining control of their breathing during stressful feelings or events.

I thought [the breathing feature] was good. I know I hear about that stuff all the time, about people breathing …it relaxes them. Sort of like meditating a little bit. Yeah, it's good.

The breathing exercises, I thought, were really good, because there are a lot of people, including myself, that have a hard time, when they're going through a hard time, can't get their breathing under control. So, pulling that up [on the screen] can really help. So I think that's good.

Oh, um, I thought [the breathing feature] was great. It helped me clear my thoughts, you know. I mean you just relax, ‘cause I was having a rough day and then it just brightened my mood, so.

##### 3.2.4.3. SquareMoves feature

The SquareMoves Feature refers to the Tetris-like game in the app where youth tap the screen to rotate blocks and fit blocks together. When a line is completed, a graphic reward occurs, and the youth score is displayed. Youth found the feature of a game in the app to be very positive in terms of visuals, ease of use, appropriate challenge, and the experience of “fun”. No youth disliked this game. Youth found that playing this game was helpful to distract from negative emotions and engage more in positive emotions by tackling the challenges (i.e., controlling the speed of the block drops) and playing in a familiar format. The most common sub-code that was found was *SquareMoves: Positive Reaction*. Youth had a positive reaction to the SquareMoves activity, which was similar to common arcade games that they had played in the past. Youth found that playing it over time could lead to relaxation.

Oh, um, it was just a relaxing game, you know. […] I just spent hours playing that game, I just zoned out.

Yeah, I liked it.

##### 3.2.4.4. Art feature

The art feature refers to the integrated drawing space on the app. The most common sub-code that was identified was *Art Feature: Positive Reaction*. All youth had a positive reaction to the art feature in the app, and no youth had a negative reaction. Youth mentioned that engaging in this feature was helpful in managing and expressing emotions. There were several different ways youth reported using this feature. Specific strategies included doodling, writing out emotional words, and drawing symbols and scenes to express their emotions. A few youth noted:

I really liked it. I can, like, doodle all the stuff. Yeah, I just really like doodling and the art option.

I think it's good. I'm not really good at art, but I think it's good for people that maybe they do art to get past their feelings, or if they're upset, they do art to calm down their nerves, or something like that. But I actually like art and stuff.

I would just go on there and write one word about how I was feeling, and that was it. So if I was feeling sad that day I would go on there and write a big capital letter ‘SAD,' and that's what I would do.

##### 3.2.4.5. Rate my mood feature

The rate my mood feature on the JoyPop™ app allows users to become more aware of their mood, starting with a query on “happiness” and then giving different negative mood options (sad and angry) and a non-committal mood (“meh”). Youth found the slide activity (sliding a wave visual up and down to reflect the mood intensity) was interesting to do. Youth liked that it was not overly complicated with emotions. They appreciated rating their different emotions across the day and reported that they could detect patterns and changes in emotions. All youth responded positively to this feature, and no youth identified negative reactions. The most common sub-code that was identified was *Mood: Positive Reaction*. Most youth enjoyed being able to name and investigate their mood and also acknowledged that their mood changed throughout the day.

Uh, I think it's good. I think it's good that, um,…. when you say that you're sad, and you can. It actually goes into detail, and you could tell it, you're actually like mad. Or how you said you're having a “meh” day or whatever. Yeah, I think it's good.

I thought that was a good one too because even myself would go on there, and it helps you feel, um, heard about what you're feeling that day because you want someone to ask you, how you're feeling today. When you don't have that, you can just go on there and rate it, and it tells you if you're in a bad mood, it tells you are you feeling okay, what's wrong. So, I think it's good for someone who needs to feel heard.

Yeah. If I have a really weird day, like a “meh” day, which I have almost like, every day, at the start [of the day]. But it turns into a happy day.

##### 3.2.4.6. Journaling feature

The journaling feature provides the user space to write down their thoughts, and seems to be a familiar activity for youth. Youth could utilize their iOS microphone options, add emojis from their phone template, or type in their text. Resilience prompts are available to write about, or youth could use the space for expressive or reflective practice. Youth reported it as a useful activity to organize their thoughts and be expressive about their emotions. All youth reported this feature as positive, and no negative reactions were identified. The most common sub-code that was found was *Journaling: Positive Reaction*. Committee members commented on this finding in terms of the value of this feature in providing a private place for self-expression. Examples from youth are:

Journal, it's good, now, you can go in there every day. You can write how you're feeling, or about how your day went. Stuff like that. It's not going to be shared with nobody, though. People write in there and stuff like that. Yeah, it's a good thing that people can write down their feelings, and how their day went and stuff.

It helped me get my thoughts out, using that. Just trying to… cause I have a lot to think about, and, yeah. It was… sometimes you gotta write what you're thinking down.

The journaling… I think it's good too. Personally, I didn't use it that much, because I have my own journaling thing I do for myself, but I think that it could be good for others.

##### 3.2.4.7. Circle of trust feature and helplines

The Circle of Trust feature allows users to input contacts of people that they trust in case of a crisis or need for support. Although this feature was highly valued as culturally consistent by committee members, youth did not use it. Youth reported not being familiar with reaching out for help outside of in-person options or ever using helplines. There was a feeling of distrust toward connecting with people who were not family, friends, or very well-known to them. The most common sub-code that was identified was *Circle of Trust: Negative Reaction*. Most youth had a negative reaction to this feature in terms of being unsure about how the information would be stored or used given the direct connection to others' contact numbers. In short, youth identified needing more information as to how their information would be protected in the app. Youth comments are as follows:

I personally, again, that one isn't for me. I can't. It's really like my trust has to stay within this area, it can't be out like that.

I didn't really know what to type there so yeah I just didn't know what to type.

When it comes to mental health for me, I have to full out talk to people. Because I'm not good with technology.

##### 3.2.4.8. Adaptations

Adaptations refer to suggestions to improve the app, with a particular focus on how to make the app more culturally relevant. Notably, the significance of cultural relevance cannot be overstated as it has been shown to have a substantial impact on the effectiveness of tools and interventions aimed at engaging Indigenous youth. Studies have demonstrated that culturally relevant interventions have found success in resonating with their intended audience and achieving desired outcomes (49, 50).

All youth had positive feedback related to the games in the app. Youth strongly favored games, which they identified as a positive way to improve feelings of happiness, and encouraged that more games be added and that game creation be a feature for Indigenous youth: “There could be more games too. As a youth, especially boys, they are more connected to games. With the sounds of it (i.e., using sounds), I feel like there can be more of those too.” Most youth had Indigenous-specific feedback to improve the app. One of the suggestions was to include an Indigenous dice game played with others called Gayendowa:neh. Another suggestion was to include Indigenous language within the app and add more Native symbols as icons, such as a wampum belt, clan animals, and feathers.

I don't really know… Just maybe add some kind of Native stuff. Like feathers or maybe clans. That would be a good thing to put in there. Put your clan and your nation in there. Maybe the animals. Like the ones that are on the earth, and the ones that are in the sky. Umm, yeah, just more Native stuff.

Most youth identified stories as an important aspect of Indigenous culture. Traditional stories were identified as something that could be integrated into the app as an activity that users could participate in, and many found these stories to be interesting and relaxing. Additionally, all youth expressed the desire to incorporate Native languages into the app and provided suggestions on how this could be achieved, such as the ability to change the language of the whole app to align with their preferred language.

…you know, I think stories could be good for the app. Putting little stories in there for people could be interesting. Just to get people's mind off things.

…for Haudenosaunee, I think there can be words added to it. Just little tiny words, expressing being happy or mad, on there, in our language.

Maybe like a setting of different languages, like if you speak a different language you can just press it, and switch it to the language that you speak.

## 4. Discussion

This study took a phenomenological approach to understand the accessibility and feasibility of the JoyPop™ iOS mobile application for Indigenous youth, partnering with a Haudenosaunee cultural and Mohawk and Cayuga language immersion school on the Six Nations of the Grand River. These findings are consistent with earlier JoyPop™ studies which found that the app includes favorable features and designs, is well-utilized on a weekly basis, and engendered perceptions of positive learning and emotion regulation. While there is no specific method or protocol to approach research with Indigenous communities, the process across this research project was consistent with established frameworks for Indigenous-oriented research (e.g., Two-eyed seeing; OCAP principles) and is a potential model conducive to continued app research and development. By partnering and collaborating with the Six Nations of the Grand River, this study underscores the importance of involving Indigenous communities directly in the research process to ensure there is greater resonance impact and planning next steps.

Given that this feedback is from a Haudenosaunee perspective, there remains interest in a Haudenosaunee-specific resilience app that may utilize the positive features found in JoyPop™ and extend in additional directions. The Good Mind concept, significant to the Haudenosaunee People, emerges as a promising thread that could be weaved, for example, more explicitly throughout all app features. This highlights that nation-specific research and resource development are important, as in other reserves, youth may connect more strongly to other cultural referents (e.g., significant historical figures and particularly animals such as fish in a coastal community and deer or bison in a land-based community). As such, a pan-Indigenous approach to a resilience app may not be consistent with all Indigenous perspectives, which may necessitate tailoring app approaches to specific cultural contexts.

Certain app features were considered unique, helpful, and engaging over time. This included the Art Feature and Breathing Feature. Art has been identified as a flexible vehicle for exploring positive and negative emotions and has been identified as a coping mechanism by Indigenous youth in the literature (51). Although the Art feature exclusively refers to drawing, the youth's positive feedback aligns with Indigenous practices and beliefs about art mediums as forms of expression, celebration, and healing (52). The Breathing feature was available for use to combat stress and negative emotionality, which was suggested in terms of ratings on overall mental health. Indigenous perspectives tend to endorse spiritual reflection and relaxation through activities like meditation (53). To align more with Indigenous youth, visual animations that utilize Indigenous teaching may be preferable to the current depiction of a circle expanding and contracting, such as symbols of trees that are honored or eagle feathers, which are used in traditional healing ceremonies (54). Given the higher overall risk for suicidality among Indigenous youth and the capacity for routine use of breathing techniques to physiologically address emotional regulation, such an activity may be important as a stand-alone activity outside of the app (33).

Youth were eager to provide recommendations to improve the app in alignment with their cultural grounding and Native languages. Integrating Indigenous cultural elements was previously emphasized among adult participants, who most frequently recommended changes in the design and layout of the JoyPop™ app (38). Participants of this study also recommended adding more games to the JoyPop™ app, but did not specify any type or specific game, suggesting that active engagement with incremental challenges can be harnessed toward resilience goals. This suggestion aligns with prior findings where the SquareMoves feature was among the most used and helpful features by providing a positive form of distraction (from negative emotions, such as panic, anxiety, and boredom). Games also play a significant role in Indigenous culture. For example, the game of lacrosse holds many important cultural beliefs and practices for Indigenous people. The Haudenosaunee believed that Lacrosse was the Creator's game and that the spirits used the game of lacrosse as a way to resolve conflicts in the Sky world (55). This game was then gifted to the Haudenosaunee, who now use lacrosse as a way to heal and strengthen community ties (55). Thus, the suggestion of adding more games to the JoyPop™ app may highlight these underlying values and beliefs, which would further support the wellness and healing of Indigenous youth. It is noteworthy that the gaming feature, as well as the journaling and art drawing features, were used by all participants most frequently, in comparison to other features (SleepEase and Circle of Trust). Although employing distraction can be a valid tool for emotional regulation, the suggestion to incorporate more games into the JoyPop™ must be considered in reference to the Indigenous principle of balance. This becomes even more significant when acknowledging the potential drawbacks associated with relying excessively on gaming for coping. Research has highlighted the risks and potential dangers associated with the overuse of distraction-based coping strategies or emotional avoidance, such as the risk of gaming addiction (56). Within the app, this is mitigated by the use of features across features to support both active reflection (Journaling) and active distraction or engagement in eye-hand coordination challenges (SquareMoves), as well as being able to rate and label emotions.

Finally, we were surprised to find the lack of resonance among the youth with the Circle of Trust feature. Previous research with Indigenous adults reported that the feature was most praised as it aligned with Indigenous values of maintaining relationships, and the pre-test similarly found that all participants valued relationships (38). This finding led to the exploration of the underlying reasons behind this lack of resonance and uncovered valuable insights that relate to the historical and socio-cultural context of colonization and technology. The negative reactions from youth and their unease around the privacy of the app might signify a broader mistrust of technology that has been inherited from historical experiences of colonization. Reactions from the youth toward the Circle of Trust feature ranged from confusion regarding usage to concerns about privacy and a sense of distrust toward person-specific information. One possible solution would be to address these issues through improved instructions. However, these responses from youth, more importantly, shed light on the remaining ties between technology and colonization, which tend to position technology as having Western-European ontologies and the legacy of unethical research practices. Technologies are often associated with unequal power dynamics, cultural assimilation, and the legacy of unethical research practices (57).

### 4.1. Strengths, limitations, and future directions

The study process was shepherded by a variety of consultations and ongoing commitment from the Six Nations community, which serves as the study's strength. Community members prioritized youth safety in the sequencing of research studies, and adaptations were made to ensure adherence to community guidelines (e.g., having a trusted member of the school community recruit the youth, as typically, a research assistant would recruit to avoid coercion). By adapting protocols to uphold community guidelines and preferences, this study took a community-based research approach that promoted collaboration, community wisdom, and co-ownership of research procedures (58, 59). This iterative method to the study process is particularly important when considering the history of exploitative research practices that have led to distrust.

While this research study took great care to implement collaborative community-driven research, there are other ways researchers can help rebuild trust with Indigenous communities, such as by utilizing existing Indigenous-led ethical protocols and standards that have been identified (60). Within these protocols, themes include: (1) balancing individual and collective rights, which includes discussing the intellectual property and ownership of data collected from Indigenous communities; (2) ensuring culturally grounded ethical principles. This includes incorporating a decolonized approach and the values, beliefs, and culture of the specific Indigenous community one is working with; and (3) ensuring community-driven/self-determined research so that Indigenous communities could access the data collected, analyzed, and the findings so that the research conducted could help them in further planning and secondary data analysis. The research methods and approach have to be cultivated together with collaborative discussion between communities and researchers to rebuild Indigenous peoples' trust.

A limitation of the study is its small sample size, making it difficult to draw overarching conclusions about the preferences of Indigenous youth toward the JoyPop™ application. The start of the COVID-19 pandemic prior to active data collection likely contributed to the lower number of youth participating (61). A larger sample size may provide more insight into the inconsistencies between this study and the perspectives of Indigenous adults, particularly whether the Circle of Trust feature would promote relationships in alignment with Indigenous principles or foster distrust toward the app's handling of personal information. A future pilot study with a larger sample would be relevant for evaluating the generalizability of the current themes to a broader sample of youth across Six Nations (i.e., not immersion school attendees), other locales of Six Nations (in Quebec, Ontario, Canada, or New York State, US), or those living off-reserve. In short, sub-population research is essential in the app's early development phases.

The purpose of this study was to explore the accessibility and feasibility of the JoyPop™ app with Six Nations youth and gain insight from participants on how app features can be adapted to be more relevant for its target population. Mental health mobile applications continue to hold promise as an mHealth intervention. With few resilience-oriented interventions, continued examination of app-based supports for enhancing resilience and health promotion seems resonant with Indigenous values and needs.

## Data availability statement

The datasets presented in this article are not readily available because the dataset being Indigenous is not a public database and the Six Nations of the Grand River control access to it, based on Canadian ethics guidelines for research with Indigenous persons. Requests to access the datasets should be directed to allison.ay28@gmail.com.

## Ethics statement

Ethics approval was received by Haudenosaunee Confederacy Council, Six Nations Elected Band Council's Research Ethics Committee, and McMaster Research Ethics Boards for consultation interviews and survey data collection (MREB #3728; HIREB #12572). The studies were conducted in accordance with the local legislation and institutional requirements. Written informed consent for participation in this study was provided by the participants' legal guardians/next of kin. Written informed consent was obtained from the minor(s)' legal guardian/next of kin for the publication of any potentially identifiable images or data included in this article.

## Author contributions

AA-Y: Formal analysis, Investigation, Writing—original draft, Writing—review and editing. DM: Writing—original draft, Writing—review and editing. KB: Writing—original draft, Writing—review and editing. DG: Conceptualization, Writing—review and editing. KM: Conceptualization, Methodology, Validation, Writing—review and editing. DM-H: Conceptualization, Methodology, Validation, Writing—review and editing. CW: Conceptualization, Formal analysis, Investigation, Methodology, Supervision, Validation, Writing—original draft, Writing—review and editing. TG: Writing—review and editing.

## Six Nations Youth Mental Wellness Committee

Tristan Bomberry, Lori Davis Hill, Daogyehneh Amanda General, Tehota'kerá:tonh Jeremy Green, Chase Harris, Beverly Jacobs, Norma Jacobs, Makasa Looking Horse, Dawn Martin-Hill, Cynthia Denise McQueen, Tehahenteh Frank Miller, and Jennifer Mt. Pleasant.
